# Adherence to Hunger Training over 6 Months and the Effect on Weight and Eating Behaviour: Secondary Analysis of a Randomised Controlled Trial

**DOI:** 10.3390/nu9111260

**Published:** 2017-11-17

**Authors:** Michelle R. Jospe, Rachael W. Taylor, Josie Athens, Melyssa Roy, Rachel C. Brown

**Affiliations:** 1 Department of Human Nutrition, University of Otago, Dunedin 9054, New Zealand; michelle.jospe@otago.ac.nz; 2 Department of Medicine, University of Otago, Dunedin 9054, New Zealand; rachael.taylor@otago.ac.nz (R.W.T.); melyssa.roy@otago.ac.nz (M.R.); 3 Department of Preventive and Social Medicine, University of Otago, Dunedin 9054, New Zealand; josie.athens@otago.ac.nz; 4 Nutrition Society of New Zealand, Whanganui 4543, New Zealand

**Keywords:** food intake regulation, hunger, obesity, blood glucose self-monitoring, adherence

## Abstract

Monitoring blood glucose prior to eating can teach individuals to eat only when truly hungry, but how adherence to ‘hunger training’ influences weight loss and eating behaviour is uncertain. This exploratory, secondary analysis from a larger randomized controlled trial examined five indices of adherence to ‘hunger training’, chosen a priori, to examine which adherence measure best predicted weight loss over 6 months. We subsequently explored how the best measure of adherence influenced eating behavior in terms of intuitive and emotional eating. Retention was 72% (*n* = 36/50) at 6 months. Frequency of hunger training booklet entry most strongly predicted weight loss, followed by frequency of blood glucose measurements. Participants who completed at least 60 days of booklet entry (of recommended 63 days) lost 6.8 kg (95% CI: 2.6, 11.0; *p* < 0.001) more weight than those who completed fewer days. They also had significantly higher intuitive eating scores than those who completed 30 days or less of booklet entry; a difference (95% CI) of 0.73 (0.12, 1.35) in body-food choice congruence and 0.79 (0.06, 1.51) for eating for physical rather than emotional reasons. Adherent participants also reported significantly lower scores for emotional eating of −0.70 (−1.13, −0.27). Following hunger training and focusing on simply recording ratings of hunger on a regular basis can produce clinically significant weight loss and clinically relevant improvements in eating behaviour.

## 1. Introduction

Why we eat, as well as what we eat, influences body weight [1]. For example, emotional eating or eating in response to negative emotions has been found to predict later obesity [1,2]. On the other hand, intuitive eating, where an individual tends to eat in response to their appetite cues may be an effective way of managing body weight, particularly in the face of societal and environmental pressures to eat in the absence of hunger. Higher intuitive eating scores have consistently been associated with lower body mass index (BMI) and better psychological health in observational analyses [3,4,5,6,7]. However, interventions that teach intuitive eating and appetite awareness have generally not resulted in weight loss to date [7,8,9,10,11]. One possible explanation may be that some overweight and obese individuals have blunted sensations of hunger and satiety [12,13,14], which may lead to difficulties in learning to eat to appetite. One solution may be to use an objective biomarker of hunger, such as blood glucose concentrations, even though it is only weakly associated with perceived hunger [15,16]. Importantly, blood glucose monitors are affordable and widely available due to the high global prevalence of diabetes [17].

However, training individuals to eat only when hungry on the basis of blood glucose measurements has been tested in only two studies [18,19]. Ciampolini et al. [20] reported significantly greater weight loss (difference of 3.5 kg) over 5 months in participants who were only able to eat when their blood glucose was below a standard cut-off (4.7 mmol/L). As we had concerns regarding the feasibility of participants being able to restrict food intake to only when blood glucose was this low, we undertook a feasibility study, which demonstrated that adherence to the protocol was greater over two weeks if an individualized blood glucose cut-off was used (determined from fasting blood glucose) [19]. In our subsequent randomized controlled trial (RCT), we examined the efficacy of this modified protocol for producing weight loss over 12 months in conjunction with diet and exercise advice [21]. We observed a similar degree of weight loss as that observed in the previous trial (3.9 vs. 3.5 kg, respectively) [20,21], although our difference did not reach statistical significance. Regardless, hunger training did produce significantly greater weight loss than other types of monitoring strategies (brief support and diet monitoring) and was rated as a very helpful strategy for weight management by our participants [21].

Examining how adherence to hunger training might affect health outcomes seems warranted, given the potential for this relatively simple method to influence weight. Measuring adherence to an intervention helps us to understand what components are having the greatest influence on outcomes and provides useful information for up-scaling an intervention in a public or primary health setting [22]. Furthermore, while hunger training aims to reduce emotional and external eating and increase intuitive eating, the impact of hunger training on eating behaviour has not been investigated. Therefore, the aims of this paper were to (i) identify which measures of adherence are most predictive of weight loss with hunger training, and (ii) examine how adherence to hunger training over six months affected weight and eating behaviour.

## 2. Materials and Methods

### 2.1. Study Design

This was an exploratory secondary analysis of the SWIFT (Support strategies for Whole-food diets, Intermittent Fasting, and Training) study, which was a five-arm parallel RCT that examined the effect of different monitoring strategies on weight and health over 12 months [21]. This analysis specifically examined the hunger training arm (*n* = 50) [23]. As a protocol paper for the wider study has been published [23], only necessary details will be provided here. Participants (250 adults, over 18 years of age, BMI ≥ 27 kg/m^2^, living locally) were recruited between November 2014 and April 2015. Participants in all groups received diet and exercise advice in one face-to-face session (30–45 min) at baseline. These were delivered by trained researchers (nutritionist, dietitian, medical doctor) who advised participants on how to follow their chosen diet (three options available) and exercise (two options). Extensive resources were provided for each diet and exercise plan, plus a comprehensive resource outlining several behavioural weight loss strategies (17). Participants were subsequently randomised to a control group (no additional monitoring support) or one of four different monitoring strategies: (i) Face-to-face monitoring (brief monthly appointments to be weighed and chat about progress), (ii) self-monitoring of daily weight (advised to weigh themselves daily with brief monthly email feedback), (iii) self-monitoring of dietary intake using MyFitnessPal (advised to enter food intake into app and/or website), and (iv) self-monitoring of hunger (hunger training, explained below). The SWIFT study was approved by the University of Otago Human Ethics Committee (H14/024) and was registered with the Australian New Zealand Clinical Trials Registry ACTRN12615000010594. All participants provided written informed consent.

### 2.2. Participants

Between November 2014 and April 2015, 250 participants were recruited based on the following inclusion criteria: living in Dunedin, New Zealand; were at least 18 years of age; had a BMI greater than 27 kg/m^2^ and fasting blood glucose less than 7 mmol/L; were not pregnant or breastfeeding or planning to conceive in the next 2 years; did not have a diagnosis of diabetes, endocrine disorders, cardiovascular disease or other serious medical condition; and were not taking medication that affected weight or body composition.

### 2.3. Outcome Measures

All measures were assessed by researchers blinded to group allocation. At baseline, participants completed questionnaires on demographics (age, sex, education) using relevant New Zealand census questions (stats.govt.nz/Census); the ten-item personality inventory [24], which gives broad scores for the ‘Big Five’ personality dimensions; the brief resilience scale [25], which assesses the ability to recover from stress; and the dieting and weight history questionnaire [26].

At baseline and 6 months, intuitive eating was assessed using the 23-item Intuitive Eating Scale-2 (IES-2), which measures 4 domains: body-food choice congruence (choosing foods that match physical needs); eating for physical rather than emotional reasons; reliance on hunger and satiety cues (trust in appetite cues to guide eating behaviour), and unconditional permission to eat subscale (willingness to eat when hungry and not label any foods as forbidden). IES-2 shows good internal consistency (Cronbach’s α = 0.87 and 0.89 for women and men, respectively) and stability (r = 0.88 and 0.92 for women and men, respectively) [4]. The 33-item Dutch Eating Behavior Questionnaire (DEBQ) [27], was used to evaluate emotional eating (eating in response to negative emotions), external eating (eating in response to the sight and smell of nearby food), and restrained eating (the cognitive effort to restrict food intake). Each of these scales results in a score from 1 to 5. The DEBQ shows good internal consistency, and factorial, and predictive validity, with reliabilities of 0.96, 0.85, and 0.93, respectively [28].

Weight (BC-418, Segmental Body Compostion Analyzer, Tanita, Tokyo, Japan), height (Heightronic Digital Stadiometer, QuickMedical, Issaquah, WD, USA), and waist circumference (at the narrowest point between the lower costal border and the top of the iliac crest by nonelastic Lufkin tape, Sparks, Maryland) were measured in duplicate, and in triplicate if the first two measures differed by more than 1%. BMI was calculated (kg/m^2^). Participants were measured wearing minimal clothing by trained research assistants who were blinded to intervention allocation.

### 2.4. Hunger Training Intervention

Participants had three hunger training appointments (baseline, day 7 and day 28). At the first visit, participants were introduced to hunger training and taught to measure their blood glucose. The following day the researcher telephoned the participant to ensure that the instructions were understood and could be followed, and to answer any questions. At the second visit, participants had the opportunity to ask questions and discuss challenges and successes. On the last visit, participants chose whether they wanted to continue to measure their blood glucose or to only fill out the other components of the booklet, which included hunger intensity before and after eating, type of hunger, and food consumed. They were given supplies for the remainder of the intervention.

The hunger training procedure was similar to that used for our feasibility study [19], with modifications to the frequency of monitoring to make it suitable for a longer intervention. For the first fortnight, participants were instructed to measure their capillary blood glucose from a finger prick sample by portable glucometer (Freestyle Optium Glucose Meter, Abbott, Doncaster, Australia) every time they wanted to eat (or drink a caloric beverage). Participants were then directed to eat only if their blood glucose concentration was equal to or less than their individualised cut-off (the average of their fasting glucose concentrations over two mornings). If their blood glucose was above their cut-off, they were instructed to choose an activity that distracted them from food, and to wait for new feelings of hunger for at least an hour before testing their blood glucose again. Participants were permitted to consume hypocaloric drinks (e.g., water, black tea, black coffee, diet soft drinks, etc.) at any time. Participants were also specifically instructed that they could only drink alcohol if their blood glucose was below their relevant cut-off. A fortnight was chosen, as it was shown to be the suitable duration to train the majority of participants [18]. Blood glucose monitoring was optional after the first fortnight.

Alongside the blood glucose monitoring, participants were asked to write in a booklet prior to each eating occasion. They noted the time before and after eating, hunger intensity on a 100 mm visual analogue scale (anchored with “not at all hungry” to “extremely hungry”), type of hunger (used with permission from Craving Change™), blood glucose concentration (if measured), and food eaten (only very brief details required). For type of hunger, participants chose from mouth hunger (eating for taste or pleasure); heart hunger (eating for non-physical reasons, such as emotions or learned behaviour); and stomach hunger (physical need for food). Participants were able to choose more than one type of hunger per eating occasion. Participants were asked to complete their hunger training booklets daily for the first month, and for one week per month thereafter (a total of 63 days). Participants could track more often if they wanted to.

### 2.5. Adherence to Hunger Training

Measures of adherence to hunger training were based on information gathered from the hunger training booklets and included months 1 to 6 to enable the largest possible sample size. Five indices of adherence were chosen a priori to examine which best predicted weight loss with hunger training, and are described in Table 1. These adherence measures were chosen as they examined the various aspects of hunger training. Adherence was assessed for the first fortnight of hunger training, which was intensive, and over the entire 6-month intervention. The distributions of adherence measures were examined graphically with quantile-quantile (QQ) plots against the standard normal distribution and determined to be not normally distributed. Adherence measures were therefore categorised ad-hoc based on the data distributions. Adherence measures included:

The frequency of booklet entry (measure 4) was used as a proxy for the frequency of hunger training as it was the minimal requirement to be classified as a hunger training day.

### 2.6. Statistical Analysis

All analyses were performed using R [29] with all statistical tests performed at the two-sided, 0.05 level. The analysis was based on intention to treat, with participants included in the analyses if they returned at least one hunger training booklet. Differences in dropout rates were calculated using chi-squared and *t*-tests, and differences in weight loss were calculated using *t*-tests. Linear regression models were used to model adherence measures on amount of weight loss, and eating behaviour outcomes, which were all adjusted for sex. As age and baseline weight had no significant influence on weight loss, they were excluded from the multivariate analyses. To identify which adherence measures best predicted weight loss, an exploratory analysis was conducted by initially entering adherence measures 1 through 5 into the bivariate models with sex, and those with *p* > 0.2 were included in the multivariate model. Backwards selection was used to yield the final model using Bayesian information criterion (BIC), and the model with the lowest BIC was chosen. The final model was then used to examine the effect of different levels of adherence to hunger training on weight and eating behaviours. Logistic regression was used to determine whether baseline characteristics were associated with the most predictive adherence measures. For the post-hoc analysis, we adjusted both the confidence intervals and *p*-values for multiple comparisons using the Westfall method [30].

## 3. Results

### 3.1. Participants

Table 2 shows that the participants were predominantly European, middle-aged, and university-educated, with an average BMI of 33 kg/m^2^ at baseline. Men constituted 38% of participants. There were no significant differences between the entire sample and those who were included in the 6-month analysis.

From an initial 50 participants, 36 (72.0%) completed the 6-month and 27 (54.0%) the 12-month assessment, with no difference in dropout rates between 0 to 6 and 6 to 12 months (*p* = 0.950). Analysis of hunger training adherence included 34 participants, as 2 of the 36 participants who were weighed at 6 months did not return their hunger training booklets.

### 3.2. Weight Loss

On average, participants in the hunger training arm lost 4.9 kg (95% CI: 3.1 kg, 6.7 kg; *p* < 0.001) between 0 and 6 months and 5.3 kg (95% CI: 2.8 kg, 7.8 kg; *p* < 0.001) between 0 and 12 months. There was no significant difference in weight loss between 0–6 months and 7–12 months (0.05 kg difference, 95% CI: −1.4 kg, 1.5 kg; *p* = 0.941). We therefore restricted our analyses to the 0–6 month period to enable the largest sample size possible.

### 3.3. Blood Glucose Monitoring

The average blood glucose cut-off (the minimum blood glucose concentration at which the participants could eat for the first fortnight) was 5.66 mmol/L (SD 0.66 mmol/L). As per our feasibility study [7,8,9,10,11], adherence to blood glucose monitoring was measured in terms of the percentage of times blood glucose was measured prior to eating, and compliance to eating only when below the blood glucose cut-off during the first fortnight. The within-person proportion for measuring blood glucose before eating was 89.0% (SD 25.7%) of eating occasions; and the proportion for only eating when blood glucose was under the individualised cut-off was 75.7% (SD 18.7%) of eating occasions.

Participants were asked to measure their blood glucose before every eating occasion during the first fortnight, after which it was optional. All 34 participants measured their glucose during the first fortnight, with an average of 43.5 (SD 12.5) times for the group (approximately 3.1 times per day for 14 days). Twenty-nine participants (85.3%) continued to measure their blood glucose beyond the first fortnight, and lost 5.6 kg (95% CI: 0.8 kg, 10.3 kg; *p* = 0.21) more weight than those five participants that stopped measuring their blood glucose after two weeks.

### 3.4. Adherence to Hunger Training Booklet

**Figure 1:**
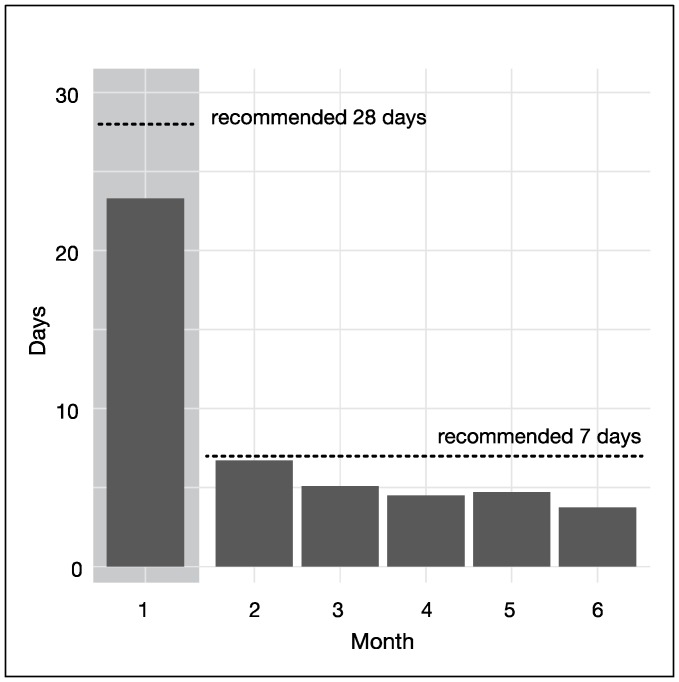
Adherence to filling in hunger training booklets over 6 months.

The number of days participants recorded at least one item in their booklets ranged from 7 to 132 days, with an average of 48.0 days (SD 27.6 days). This represents 76.2% of the recommended 63 days, and was used as a proxy for frequency of hunger training. When frequency of booklet entry was categorised based on distribution, 13 participants completed 0–29 days of hunger training, 6 completed 30–59 days, and 15 completed at least 60 days. As shown in Figure 1, frequency of filling in the booklet decreased over time from 23.3 days (SD 7.6) (83.2% of the recommended 28 days) to 3.7 days (SD 5.7) (64.9% of the recommended 7 days).

### 3.5. Hunger Training Adherence and Weight Loss

Using bivariate regression models controlling for sex, we found that weight loss was significantly associated with adherence measures 3 (the frequency of measuring blood glucose) and 4 (the frequency of filling in the hunger training booklet). By contrast, adherence measures 1, 2, and 5 were not significant predictors of weight loss (all *p* > 0.06). For the final model with weight change as the dependent variable, there was no significant difference between the regression model that included adherence measures 3 and 4 (along with sex) and the model that only included adherence measure 4 and sex. Furthermore, the BIC indicated that the simpler model was superior. Therefore the final model includes the frequency of filling in the hunger booklet (adherence measure 4) and sex (Table 3). Similar findings were obtained using weight change at 12 months (Table S1).

Participants who measured their blood glucose 51–99 times lost 4.9 kg more weight (95% CI: 1.1 kg, 8.8 kg; *p* = 0.018), and those who measured their blood glucose 100 times or more lost 6.4 kg more weight (95% CI: 1.1 kg, 11.7 kg; *p* = 0.026) than participants who measured their blood glucose 50 times or less.

**Figure 2:**
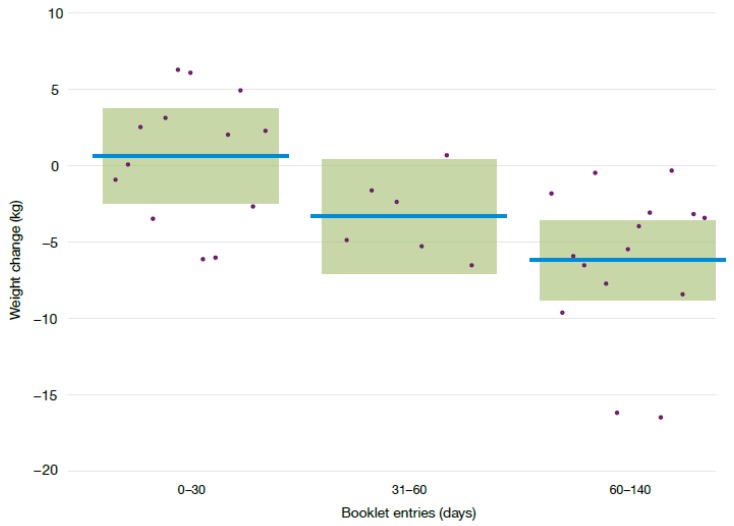
Comparison of weight loss (kg) of participants by the number of booklet entries, adjusted by sex. Horizontal lines represent the adjusted mean weight difference, the boxes represent the 95% confidence intervals around the mean values and dots represent individual weight changes.

The frequency of filling in the hunger training booklet significantly predicted weight loss. Participants who completed 60 or more days lost 6.8 kg (95% CI: 2.6 kg, 11.0 kg; *p* < 0.001) more weight than either of the less frequent groups (Figure 2). There was no significant difference in weight loss between participants who completed 0–29 days and those who completed 30–59 days (−4.0 kg difference, 95% CI: −9.6 kg, 1.7 kg; *p* = 0.093). The changes in percentage weight loss mirrored changes in BMI and waist circumference (Table 4). Men and women who filled in the hunger training booklet for 60 days or more lost 12.7 kg (95% CI: 3.1 kg, 22.3 kg) and 5.2 kg (95% CI: 3.1 kg, 7.4 kg), respectively. Further analyses showed that adjusting for baseline weight did not alter the outcomes (data not shown). Corresponding figures for weight loss at 12 months for those who completed the hunger training booklet for 60 days or more, were −11.9 kg (95% CI: −25.6 kg, 1.7 kg,) in men and −5.5 kg (95% CI: −7.8 kg, −3.1 kg,) in women.

### 3.6. Hunger Training Frequency and Eating Behaviours

Intuitive eating scores are shown in Table 5. Participants who completed at least 60 days of hunger training showed increases in two intuitive-eating domains for which higher scores indicate more intuitive eating—body-food choice congruence (0.73, 95% CI: 0.12, 1.35; *p* = 0.017), and eating for physical rather than emotional reasons (0.79, 95% CI: 0.06, 1.51; *p* = 0.032)—compared to those who completed less than 30 days. In contrast to the other intuitive-eating subscale, participants who completed 60 days or more had a decrease in the unconditional permission to eat subscale (−0.68 95% CI: −1.28, −0.09; *p* = 0.022) compared with those who completed less than 30 days.

As Table 6 indicates, participants who completed 30–59 days of hunger training had a decrease in emotional eating (−0.65 95% CI: −1.22, −0.09; *p* = 0.008) and an increase in dietary restraint (0.85 95% CI: 0.13, 1.57; *p* = 0.018) compared with those who completed less than 30 days. Participants who completed at least 60 days had a decrease in emotional eating (−0.70, 95% CI: −1.13, −0.27; *p* < 0.001) compared to those who completed less than 30 days.

### 3.7. Types of Hunger

Participants reported that they ate when they were stomach hungry most of the time (83%, SD 16% of eating occasions). Participants indicated that they were mouth hungry on 19% (SD 20%) and heart hungry on 16% (SD 16%) of eating occasions. As more than one type of hunger could be selected for a given meal, the type of hunger tally is over 100%.

### 3.8. Predictors of Adherence

Demographic characteristics, including sex (*p* = 0.602), age (*p* = 0.288), education (*p* = 0.619), or age of first dieting episode (*p* = 0.616), were not related to frequency of hunger training. Similarly, baseline questionnaire results did not predict adherence to hunger training, including how intuitively they ate at baseline (*p* = 0.836), how resilient they were (*p* = 0.994), or any of the ‘Big Five’ personality dimensions (all *p* ≥ 0.117).

## 4. Discussion

A relatively simple and feasible intervention designed to train individuals to eat only when truly hungry produced clinically significant weight loss and beneficial changes to eating behaviours—as long as adherence criteria were met. Frequency of booklet entry was the most important adherence measure and participants had to adhere by simply recording a single item (e.g., type of hunger, blood glucose concentration, whether food was eaten or not) approximately one-third of the time (minimum required adherence was 60 of 180 days). While this seems a relatively low burden for participants given the significant weight loss produced (~13 kg in males and 5 kg in females), the level of adherence required was unattainable for the majority of participants.

Interestingly, while most interventions that teach intuitive eating and appetite awareness through subjective assessment of hunger and satiety do not result in clinically important weight loss [7,8,9,10,11], we found that our participants were able to lose weight, as did Ciampolini et al. [20]. It is feasible that this difference arises due to the use of blood glucose monitoring, which allowed individuals who may have diminished perception of hunger and satiety, to gain greater awareness of their appetite signals. Measuring blood glucose, at least in the first fortnight, is likely an important element in hunger training, and our results show that measuring blood glucose at least 100 times (approximately 7 times per day if only measured in the first fortnight) did result in significantly greater weight loss. Moreover, although intuitive eating interventions haven’t been successful for weight loss, they do appear to be beneficial for weight maintenance, particularly over the longer term (18 months or more) [31,32,33]. The combination of the low contact required for hunger training and the potential for sustainable weight management makes it a promising treatment for primary and public health dissemination.

The men in our study lost more weight than women, which supports some [34,35], but not all [36] previous findings. Men are often underrepresented in weight-loss studies [37], even though their obesity rates are similar to those of women [38,39]. This lack of evidence means we know less about how men react to weight loss interventions than women. In particular, it is not known how successful interventions that address intuitive eating might be in men, since trials have only been conducted in women [8,9,10,31,33]. Our findings would suggest that hunger training may provide a particularly effective weight-loss option for men.

Results from this study further confirm that hunger training with blood glucose testing is a feasible intervention strategy [19], when combined with diet and exercise advice. Adherence measures met the feasibility criteria created in our initial study, where adherence to measuring blood glucose before eating had to be more than 80%, and adherence to waiting to eat until blood glucose was below a specified cut-off had to be greater than 75%. In this study the average blood glucose cut-off was 5.7 mmol/L, which was higher than the original protocol recommendation of 4.7 mmol/L [18], and confirms that a higher cut-off does not compromise the results of hunger training. Interestingly, over 85% of participants chose to continue to monitor their blood glucose beyond the mandatory initial fortnight, which resulted in greater weight loss. Future studies should consider measuring blood glucose for a longer duration.

Those who adhered to 60 days or more of hunger training showed substantial decreases in non-hungry eating and an increased reliance on appetite to guide food intake (with all changes between 0.5 to 2 SD in size), which would be considered beneficial changes to eating behaviour [4,40]. On the other hand, dietary restraint scores increased, albeit only in one questionnaire (IES-2) and not the other (DEB-Q). Dietary restraint is the cognitive effort to restrict food intake and is complex to interpret because it is implicated in both healthy (e.g., effective weight management in an obese individual) and detrimental (e.g., binge eating) outcomes. Crucially, dietary restraint can assist in weight loss without increasing the risk of disordered eating patterns when it is used with consistent self-monitoring and realistic weight management goals [41], as occurs in hunger training.

We found that 36% of participants were able to adhere to hunger training in such a way that it yielded clinically relevant outcomes for weight; however, we were not able use baseline characteristics to predict which participants would adhere the most. Difficulties in adhering to treatments for chronic diseases are commonly reported in the literature [22]. It is unlikely that the same treatment is effective for everyone suffering the same disease, since adherence is influenced by many factors that interact in ways not yet understood [22,42]. Unfortunately, it is not yet possible to predict a person’s adherence to a treatment, as no personality traits have been reliably associated with adherence [22], which is consistent with our results. Finding ways to increase adherence has been thus far neglected in research, and merits investigation, especially as it is clear from this intervention that ‘one-size does not fit all’ for obesity treatment [43]. It would be worthwhile investigating strategies for increasing adherence to filling in the booklet, including digitising the booklet and adding electronic reminders [44].

The main strength of this secondary analysis is that we are able to provide a recommended dose of hunger training of 60 days in 6 months, which will be useful for future interventions and implementations. This study also confirms that hunger training is feasible and effective over a 6-month time frame. However, our study also has some limitations. This is a secondary data analysis as our original SWIFT study was not specifically designed to examine adherence levels to hunger training. We chose to examine the effect of adherence on weight loss at both 6 and 12 months, and observed broadly comparable results at both time points. The small number of participants included in our analysis, particularly at 12 months, merits caution when interpreting the results, but confidence intervals have been provided to show the range of possible effects. We assumed that participants were missing at random; however, there may have been unidentified biases that influenced whether a participant completed hunger training. Given our sample size, we were not able to include all variables in a multivariable model; instead, we conducted an exploratory analysis using bivariate analysis to determine potentially important predictors. Future studies should include more participants to ensure the study is adequately powered, and to allow nuanced identification of which participants will adhere to and benefit from hunger training. We relied on participants returning their booklets, which may have underestimated adherence. Being able to gather the hunger training data remotely, ideally in a time-stamped fashion, would have led to a more objective measure of adherence. At present, this is not feasible; however, with advancing technology in self-monitoring and non-invasive glucose measurement, along with mobile app development, this may become a possibility in the near future [45,46].

## 5. Conclusions

Overall, results from this secondary analysis indicate that hunger training is a feasible and effective method for losing weight and improving eating behaviours among adults, as long as adherence guidelines are met. Participants and patients should be encouraged to measure blood glucose consistently for at least two weeks, and to use the booklet daily for one month followed by one week per month to achieve clinically important health benefits. For the one-third of participants who can adhere, hunger training seems to be an effective weight loss strategy, providing promise as a primary and public health obesity treatment. Elucidating how best to encourage adherence so that these benefits could be obtained by a greater number of people would greatly enhance the applicability of this method.

## Figures and Tables

**Table 1:** Adherence measures used as predictors in hunger training.

Measure Number	Adherence Measure (Predictors)	Time	Calculation	Categories
1	Blood glucose measurement before eating	week 1–2	Eating occasions where blood glucose was reported (%)	0–70%, 70.1%–90%, 90.1%–100%
2	Eating only when blood glucose measurement was under cut-off	week 1–2	Eating occasions where blood glucose was below the assigned cut-off divided by the total number of eating occasions with a noted blood glucose values (%)	0–70%, 70.1%–90%, 90.1%–100%
3	Number of blood glucose measurements	month 1–6	Total blood glucose measurements	0–49, 50–99, 100+
4	Number of booklet entries	month 1–6	Number of days a participant recorded at least one item in the hunger training booklet ^1^	0–29, 30–59, 60+
5	Type of hunger (mouth, heart, stomach)	month 1–6	Days that the specific type of hunger was selected divided by the total number of days with a type of hunger selected (%)	0–20%, 20.1%–40%, 40.1%–60%, 60.1%–80%, 80.1%–100%

^1^ Out of a recommended 63 days, which was calculated from 28 days in month one, and 7 days each from months two to six.

**Table 2:** Baseline characteristics of the samples.

Characteristic	Entire Sample	Completers	*p*-Value
***n***	50	34	
**Age (years)**	40.8 (10.9)	42.0 (10.3)	0.637
**Female *n* (%)**	31 (62%)	21 (62%)	0.595
**University degree *n* (%)**	27 (54%)	18 (53%)	1.000
**Ethnicity (%)**			1.000
**European**	45 (90%)	31 (91%)	
**Other**	5 (10%)	3 (9%)	
**Partnered (%)**	43 (86%)	31 (91.2%)	0.707
**Weight (kg)**	95.9 (17.0)	95.3 (17.5)	0.865
**Height (cm)**	170.3 (9.5)	170.2 (9.2)	0.982
**BMI (kg/m^2^)**	33.0 (4.3)	32.7 (4.3)	0.798
**Waist (cm)**	100.3 (12.9)	100.2 (14.6)	1.000
**Fasting glucose (mmol/L)**	5.46 (1.09)	5.55 (0.74)	0.613

Values are mean (standard deviation) unless otherwise indicated.

**Table 3:** Coefficients of the multiple regression model examining the effect of frequency of booklet entry, controlling for sex, on weight change (kg) at 6 months.

Variable	Point Estimate	95% CI ^1^		*p*-Value ^1^
		Lower	Upper	
(Intercept)	0.6			
Sex (male vs. female)	−4.7	−8.1	−1.4	0.007
Frequency of booklet entry: 30–59 days ^2^	−4.0	−9.6	1.7	0.093
Frequency of booklet entry: 60–140 days ^2^	−6.8	−11.0	−2.6	0.001

Multiple *R*-squared: 0.406, adjusted *R*-squared: 0.347. ^1^ Adjusted for multiple analysis using the Westfall method. ^2^ Against the 0–30 days group.

**Table 4:** Effect of frequency of hunger training on weight, body mass index (BMI), and waist circumference.

Variable	*n*	Frequency Days	Month 0 Mean (SD)	Month 6 Mean (SD)	Difference ^1^ Mean (95% CI)
Weight (kg)	13	0–29	96.3 (16.2)	94.4 (16.0)	
	6	30–59	89.5 (14.7)	85.4 (11.5)	−4.0 (−9.6, 1.7)
	15	≥60	96.7 (20.1)	89.1 (17.3)	−6.8 (−11.0, −2.6)
BMI (kg/m^2^)	13	0–29	32.3 (4.4)	31.7 (4.4)	
	6	30–59	32.1 (2.2)	30.7 (1.4)	−1.2 (−3.0, 0.5)
	15	≥60	33.3 (4.8)	30.8 (4.6)	−2.2 (−3.5, −0.8)
Waist (cm)	13	0–29	100.2 (11.7)	97.6 (12.0)	
	6	30–59	96.2 (11.4)	93.2 (7.0)	−2.0 (−9.0, 5.0)
	15	≥60	101.9 (18.0)	95.5 (14.5)	−4.7 (−10.0, 0.5)

^1^ Difference refers to frequency group relative to 0–29 days (reference group). Adjusted for sex and adjusted for multiple comparisons using the Westfall method [30].

**Table 5:** Effect of frequency of hunger training on intuitive eating using the IES-2 questionnaire.

Variable	*n*	Frequency Days	Month 0 Mean (SD)	Month 6 Mean (SD)	Difference ^1^ Mean (95% CI)
Overall Score	13	0–29	3.03 (0.48)	2.93 (0.55)	
	6	30–59	3.02 (0.52)	3.06 (0.33)	0.09 (−0.50, 0.67)
	15	≥60	3.06 (0.49)	3.37 (0.41)	0.37 (−0.08, 0.82)
Body-food choice congruence	13	0–29	3.38 (0.78)	3.19 (0.88)	
	6	30–59	3.22 (0.34)	3.17 (0.94)	0.29 (−0.52, 1.11)
	15	≥60	3.40 (0.71)	3.89 (0.80)	0.73 (0.12, 1.35)
Unconditional permission to eat	13	0–29	3.24 (0.69)	3.38 (0.61)	
	6	30–59	3.42 (0.38)	2.94 (0.61)	−0.75 (−1.53, 0.04)
	15	≥60	3.37 (0.54)	2.87 (0.65)	−0.68 (−1.28, −0.09)
Reliance on hunger and satiety cues	13	0–29	2.78 (0.81)	2.69 (0.87)	
	6	30–59	2.98 (0.75)	2.92 (0.35)	−0.06 (−1.13, 1.00)
	15	≥60	2.78 (0.67)	3.44 (0.78)	0.68 (−0.13, 1.49)
Eating for physical rather than emotional reasons	13	0–29	2.92 (0.89)	2.69 (1.02)	
	6	30–59	2.67 (0.97)	3.21 (0.40)	0.74 (−0.22, 1.70)
	15	≥60	2.91 (0.84)	3.50 (0.75)	0.79 (0.06, 1.51)

^1^ Difference refers to frequency group relative to 0–29 days (reference group). Adjusted for sex and adjusted for multiple comparisons using the Westfall method.

**Table 6:** Effect of frequency of hunger training on eating behaviours as measured by the Dutch Eating Behaviour Questionnaire.

Variable	*n*	Frequency Days	Month 0 Mean (SD)	Month 6 Mean (SD)	Difference ^1^ Mean (95% CI)
Emotional Eating	13	0–29	2.54 (0.82)	2.75 (1.01)	
	6	30–59	2.66 (1.00)	2.36 (0.44)	−0.65 (−1.22, -0.09)
	15	≥60	2.43 (0.77)	2.01 (1.01)	−0.70 (−1.13, −0.27)
Restraint	13	0–29	2.58 (0.62)	2.55 (0.60)	
	6	30–59	2.55 (0.39)	3.17 (0.32)	0.85 (0.13, 1.57)
	15	≥60	2.68 (0.57)	2.87 (0.65)	0.33 (−0.22, 0.88)
External Eating	13	0–29	3.43 (0.75)	3.50 (0.77)	
	6	30–59	3.28 (0.35)	3.18 (0.76)	−0.25 (−0.89, 0.39)
	15	≥60	2.97 (0.71)	2.65 (0.60)	−0.43 (-0.92, 0.06)

^1^ Difference refers to frequency group relative to 0–29 days (reference group). Adjusted for sex and adjusted for multiple comparisons using the Westfall method [30].

## Supplementary Materials

The following are available online at http://www.mdpi.com/2072-6643/9/11/1260/s1. Table S1: Coefficients of the multiple regression model examining the effect of frequency of booklet entry, controlling for sex, on weight change (kg) at 12 months.
