# Supplementary material for: Adherence to Hunger Training over 6 Months and the Effect on Weight and Eating Behaviour: Secondary Analysis of a Randomised Controlled Trial
Source: Nutrients. 2017 Nov 17;9(11):1260. doi: 10.3390/nu9111260 (PMC5707732; doi:10.3390/nu9111260)
Supplement: Supplementary file 1 [file nutrients-09-01260-s001.pdf]

Table S1. Coefficients of the multiple regression model examining the effect of frequency of booklet entry, controlling for sex, on weight change (kg) at 12 months.

Variable	Point estimate	95% CI [1]		p-value [1]
		Lower	Upper	
(Intercept)	1.03			
Sex (male vs female)	−5.7	−11.18	−0.14	0.045
Frequency of booklet entry: 30–59 days [2]	−2.4	−10.9	6.1	0.562
Frequency of booklet entry: 60–140 days [2]	−6.8	−12.6	−1.0	0.024

Multiple R-squared: 0.295, adjusted R-squared: 0.203. [1] Adjusted for multiple analysis using the Westfall method. [2] Against the 0–30 days group.
